# Correction: Highly selective optical sensor N/S-doped carbon quantum dots (CQDs) for the assessment of human chorionic gonadotropin β-hCG in the serum of breast and prostate cancer patients

**DOI:** 10.1039/d3ra90095a

**Published:** 2023-09-25

**Authors:** Yasmeen M. AlZahrani, Salha Alharthi, Hind A. AlGhamdi, A. O. Youssef, Shahenda S. Ahmed, Ekram H. Mohamed, Safwat A. Mahmoud, Mohamed S. Attia

**Affiliations:** a Chemistry Department, College of Science, Imam Abdulrahman Bin Faisal University P.O. Box 1982 31441 Dammam Saudi Arabia; b Chemistry Department, Faculty of Science, Ain Shams University Abbassia Cairo 11566 Egypt Mohamed_sam@yahoo.com; c Analytical Chemistry Department, The British University in Egypt El Sherouk City Cairo 11378 Egypt; d Physics Department, Faculty of Science, Northern Border University Arar Saudi Arabia

## Abstract

Correction for ‘Highly selective optical sensor N/S-doped carbon quantum dots (CQDs) for the assessment of human chorionic gonadotropin β-hCG in the serum of breast and prostate cancer patients’ by Yasmeen M. AlZahrani *et al.*, *RSC Adv.*, 2023, **13**, 21318–21326, https://doi.org/10.1039/D3RA01570J.

The authors regret that the funding information was incorrectly shown in the Acknowledgements section of the original manuscript. The corrected funding acknowledgement is as shown below.

The authors extend their appreciation to the Deanship of Scientific Research at Northern Border University, Arar, KSA for funding this research work through the project number NBU-FFR-2023-0020.

The Royal Society of Chemistry apologises for these errors and any consequent inconvenience to authors and readers.

## Supplementary Material

